# Stable individual differences in habituation and sensitization to prolonged painful stimulation are underpinned by activity in the hippocampus, amygdala and sensorimotor cortices

**DOI:** 10.1097/j.pain.0000000000003636

**Published:** 2025-07-24

**Authors:** Richard Harrison, Carien M. van Reekum, Greig Adams, Wiebke Gandhi, Tim V. Salomons

**Affiliations:** 1 University of Reading, School of Psychology and Clinical Language Sciences, Reading, UK; 2 Queen’s University, Department of Psychology, Kingston, ON, Canada

**Keywords:** Acute Pain, Sensitization, Habituation, MRI, Quantitative Sensory Testing, Amygdala, Hippocampus

## Abstract

Acute pain serves to warn an organism of potential damage. Two plausible theoretical response scenarios for prolonged painful stimulation could be hypothesised: If the organism does not sense potential harm an individual may habituate. Whereas, if harm is possible, pain sensitization maybe more probable. Examining how an individual adapts to prolonged stimulation will provide unique insight to the mechanisms underlying pain habituation and sensitisation and, potentially, a valuable perspective on the development of chronic pain. However, currently little is known about the stability of these individual differences or their underlying neural mechanisms. To address this, eighty-five participants completed an MRI session, involving a noxious stimulation task and a resting-state scan. Habituation/sensitization was operationalized as the slope of change in pain ratings across the task. Habituation was associated with increasing activity in the anterior hippocampus and amygdala over time, with sensitization associated with increasing activity in the sensorimotor cortices. These regions were then used as seeds for a resting-state functional connectivity analysis, which revealed that habituation was associated with higher connectivity between the hippocampus and ventromedial prefrontal cortex(vmPFC), and higher connectivity between sensorimotor regions and the hippocampus, amygdala and insula cortex. We have shown that habituation/sensitization to pain is a stable trait underpinned by differential activity in brain regions supporting sensory processing and appraisal. The perspective of these stable phenotypical patterns could have clinical applications and potential for improving our understanding of the development of chronic pain.

## 1 Introduction

Acute pain serves as a warning of potential harm or damage. In response to repetitive and consistent nociceptive stimulation, it may be theorised that two opposing adaptive responses are possible: Pain sensitization might occur if the source of persistent injury is perceived as a risk of damage[44]. Alternatively, when experiencing unavoidable but non-harmful repetitive stimuli, the preservation of resources expended on defensive behaviour is adaptive, thus facilitating pain habituation[68].

Experimentally, it is frequently reported that individuals habituate to pain[18,27,29,31,33,46,61,80,82,85]. However, multiple studies demonstrate the opposite(sensitization)[24,28,40,56,62]. Most studies employ group-level analyses only, simply reporting descriptives of the sample[12,45,70,85,88], or only include incomplete individual data[18,28,33,40,56,61,91]. Sample composition varies, from predominantly sensitizers[45] or habituators[33,80] to near equal splits[12,37,70,88]. This heterogeneity may merely represent differences in experimental paradigms, alternatively, the proclivity to habituation/sensitization may vary within samples systematically. Findings suggest the proportion of habituators in a sample may be stable over time[11], although these stem from a small study(n=10).

Pro- and anti-nociceptive modulatory mechanisms have been well documented[1,13,67,93], and phenotypic nociceptive profiles[99] are at the core of research investigating biomarkers of pain sensitivity and pain chronicity[26,35,65,100]. Many studies adopt single-session quantitative sensory testing(QST) techniques such as temporal summation(TS), to quantify pain sensitization[44]. Whereas other studies quantify habituation/sensitization more broadly via changes in pain sensitivity over multiple blocks of nociceptive stimulation[18,29,31,37]. Psychological characteristics, such as depression, anxiety, mindfulness and neuroticism have also been associated with pain sensitivity[36,79,81,92] and identified as phenotypic profiles for chronic pain and treatment response[19,54,58,69,85,90]. To better understand these markers, habituation/sensitization patterns need to be investigated over multiple sessions.

The neural underpinnings of acute pain perception involve a combination of higher-order cortical processes, alongside sensorimotor processing[66,67]. Prefrontal regions play an active role in pain modulation, with direct projections to opioidergic brainstem regions, such as the periaqueductal gray(PAG) and rostral ventromedial medulla(RVM)[21,73]. Additionally, regions such as the cingulate and ventromedial prefrontal cortices(vmPFC) interact with subcortical brain regions, including the hippocampus and amygdala, to implement threat-based learning, update expectations, and appraise nociceptive stimulation[48,75,104], suggesting involvement in context-dependent adaptation of responses to repetitive nociceptive stimulation.

Repetitive stimulation has been associated with decreasing activity in sensory-discriminative regions, such as the somatosensory cortex, anterior insula and supplementary motor area[12,61,70], as well as increasing activity in medial prefrontal regions[11,12]. These may represent appraisal-based modulatory processes, alongside decreases in sensory-discriminative processing. However, these studies quantified habituation by comparing brain activation during nociceptive stimulation in early vs late MRI blocks[61,70] or across days of stimulation[12]. As pain ratings were not included in their analyses, these findings may simply represent adaptation to nociception, rather than to the holistic and subjective experience of pain.

This study will examine the stability of individual differences in habituation/sensitization to repetitive nociceptive stimulation across multiple days. We further test the extent to which these individual differences reflect engagement of distinct somatosensory and cortical networks, and with well-established psychometric and sensory pain indices. The combined approaches will enhance our understanding of phenotypical profiles of pain habituation/sensitization, and mechanisms that may underlie resilience or vulnerability to persistent pain.

## 2 Methods

### 2.1 Sample

We tested ninety-five participants between the ages of 18 and 45 years. Over the course of the experiment, 10 participants were excluded from analysis due to excessive head-movement during MRI data acquisition(n=2), malfunction of the thermal stimulator(n=7) and data corruption during data transfer(n=1). This, therefore, left a final sample of eighty-five participants(44 females, M_age_=23.45, SD=4.48y). All participants were included in all analyses unless otherwise specified. Demographics are provided via supplementary A. The study was advertised via opportunistic sampling and the use of posters, email lists and social media advertisements across the University and local community. Participants were recruited to take part in 13-sessions. The focus of this current study is on data from the first 5 sessions. The following 8 sessions consisted of psychological interventions and participants were allocated to one of two groups, one focusing on pain management strategies, or a control group focusing on interpersonal relationships. The findings from the intervention portion of the study are not described in this manuscript. Neither participants, nor experimenters, were aware of their group allocation until the commencement of the 6^th^ session, and as such, are not anticipated to have any influence on the data presented below. This description is included to provide an accurate overview of participant burden across the study[50]. The a-priori sample size of 90 participants was calculated, based on the delivery of this between-groups intervention.

All participants were right-handed, and reported no historical or current chronic pain diagnoses, neurological disorders or neuropathic conditions. Participants were asked to abstain from pain medication on the day of a session, were all right-handed and had no MRI counterindications. The study was approved by the ethics committee of the University of Reading(UREC14/04). All participants provided fully informed written consent and received reimbursement(£10/hour) for their participation.

### 2.2 Materials

#### 2.2.1 Equipment

Thermal nociceptive stimulation was administered using a Medoc Pathway ATS device[9]. The thermode was a 30x30mm Peltier thermode.

For use in the MRI, the Medoc Pathway system was fitted with an MR-filter, to reduce the influence of extraneous noise within the MR environment. Experimental stimuli in the scanner were delivered using E-Prime 3 (Psychology Software Tools, Pittsburgh, PA), where responses were provided via a fibre-optic, 4-button response pad.

#### 2.2.2 Questionnaires

To investigate the psychological features of pain habituation/sensitization profiles, participants completed the Big Five Inventory (BFI;[34]), Beck’s Depression Inventory (BDI;[9]), State-Trait Anxiety Inventory (STAI;[87]) and Five Factor Mindfulness Questionnaire (FFMQ;[7]), which were collected during sessions 3 (BFI & BDI) & 4 (FFMQ). The BFI is a 44-item inventory which captures extraversion (vs introversion), agreeableness (vs antagonism), conscientiousness (vs lack of direction), neuroticism (vs emotional stability) and openness (vs closedness to experience). The resulting score is subdivided across these five subscales. The BDI is a 21-item scale that measures symptoms of depression. A high score on the BDI represents higher symptoms of depression. The STAI is a 20-item scale measuring situational symptoms of anxiety. A high score on the STAI represents higher anxiety symptoms. Lastly, the FFMQ is a 39-item questionnaire based on a factor analysis of 5 independently developed mindfulness questionnaires. This measure provides a total score for trait mindfulness (higher scores represent higher mindfulness) across five subscales: openness, description, acceptance, non-judgementalness and non-reactivity. For the purposes of accurately describing participant burden, participants also completed the Pittsburgh Sleep Quality Index (PSQI), the BEM Sex Inventory Role (BSRI), Behavioural Activation and Behavioural Inhibition Scale (BIS-BAS), Pain Catastrophising Scale (PCS), Emotional Regulation Questionnaire (ERQ) and COPE Inventory. However, these questionnaires were not included in any of these current analyses. Within this sample, the BDI (α=.84), STAI (α=.87) and FFMQ (α=.87) demonstrated high stability and consistency. This was also the case for each of the five subscales of the BFI, extraversion (α=.85), agreeableness (α=.74), consciousness (α=.83), neuroticism (α=.85) and openness (α=.75).

Pain ratings were provided on an 11-point numerical rating scale (NRS), ranging from 0(no pain) to 10(extremely painful). Participants were instructed that, regardless of whether they can feel heat or not, if they do not consider the stimulus to be painful, they should provide a 0. Participants were also informed that they could provide ratings in intervals of 0.5 along this scale.

#### 2.2.3 MRI Acquisition

Brain images were acquired using a 3-Tesla Siemens Magnetom Prisma (Siemens, Erlangen, Germany) and all images were acquired using a 64-channel head and neck coil. The narrow size of the coil restricted head movement, although in the instance of smaller head sizes, additional foam padding was used to restrict movement. The scanning protocol consisted of anatomical and functional imaging, all of which utilised an interleaved spatial order acquisition. A T1-weighted inversion recovery fast gradient echo-high resolution anatomical scan (TR=2.3s, TE=2.29ms, FA=8°, voxel size=0.9x.0.9x0.9mm, 256x256x192 matrix), T2*-weighted gradient echo planar imaging(EPI) resting-state sequence(TR=2.29s, TE=36.4ms, FA=84°, volumes=210, multiband acceleration factor=2, voxel size= 2.1x2.1x2.1mm, slice thickness=2.1mm, matrix=84x84x58) and four EPI pain stimulation blocks(TR=2.29s, TE=36.4ms, FA=84°, volumes=168, multiband acceleration factor=2, voxel size=2.1x2.1x2.1mm, slice thickness=2.1mm, matrix=84x84x58) were collected for each participant. For the purposes of creating field maps, two spin-echo (SE) EPI scans in opposite encoding directions (PA;AP) were completed prior to the four pain EPI blocks.

### 2.3 Procedure

#### 2.3.1 Baseline Assessment (Session 1)

All participants attended an initial baseline session, wherein they were briefed on the study timeline, provided informed consent, and completed a quantitative sensory testing (QST) battery. Prior to the initiation of QST, participants were given an overview of the Medoc Pathway system (Medoc Medical Systems, Ramat Yishai, Israel), to reduce situational anxiety towards the equipment. Participants were also given a description of the NRS that they would be using to provide pain ratings when prompted. The thermode was placed in a custom-made wooden leg rest and placed next to the participant’s chair. The mid-point between ankle and knee, on the underside of the leg, was then measured by the experimenter, and participants then positioned this point over the stimulus. To ensure constant pressure, as well as that exerted by the weight of the leg, weighted beanbags were placed on the leg to keep it in position and to alert the experimenter to any movement via the sound of the beanbags being moved. Participants were instructed to keep their leg flat against the thermode, and to inform the experimenter if they lifted or moved their leg at any point.

Firstly, participants completed a pair of tasks to determine their pain threshold, quantified by averaging the results of 1) a staircase/method of levels experiment and 2) a method of limits experiment. This threshold was not used at any point within data analysis and was simply used as a starting point for further stimulus calibration. As such, description of this procedure can be found in full in previous publications[36] and are not repeated here. Based on the results of piloting, for stimulus calibration, the initial destination temperature was set as threshold+0.5°C. This calibration procedure consisted of a single 20s ramp and hold stimulus, with ramp and return rates of 8°C/s. After the administration of the stimulus (post-ramp down), the participant provided a pain intensity rating using the 11-point NRS. To reduce the risk of ceiling (intolerably painful) or floor (non-painful) effects, the optimal temperature rating was set at 5/10 (+/- 1). Therefore, if the participant rated the stimulus between 4-6, the temperature was formalised as their test temperature. If the participant rated outside of this range, the temperature was altered +/- 0.5°C, and the test was repeated until a rating between 4-6 was provided.

The participant then completed experiments to quantify temporal summation (TS), as well as conditioned pain modulation and intrinsic attention to pain tasks (not reported here). For TS, a phasic design was used, which consisted of a 120s ramp and hold stimulus, using the calibrated test temperature, with ramp and return rates of 8°C/s. This methodology was selected following recommendations for the use of tonic heat paradigms for the quantification of TS[89]. Participants were asked to provide pain intensity ratings, when prompted, at 10s intervals, with the first rating provided once the thermode reached destination temperature. This ultimately provided a total of 12 ratings. Importantly, TS is distinct from the habituation/sensitization slopes, which are the primary focus of the study (see paragraph below for more detail). TS is retained as an auxiliary variable to explain variance across the group in the habituation/sensitization slopes over time.

Lastly, the stimulus for the repetitive nociception paradigm for pain habituation/sensitization slopes (described below), was calibrated. A higher intensity of 6-8 was targeted, to allow a greater potential movement for decreasing ratings related to pain habituation, which are considered to be unlikely within the quantification of TS[89]. The calibration consisted of three 20s stimuli, with a destination temperature of threshold + 1°C. Participants provided a pain intensity rating at the end of each of the stimuli. If all three ratings were between 6-8, the habituation stimulus was set. If they rated outside of this range, the temperature was altered by +/-0.5°C, and the test was repeated until acceptable ratings were received.

#### 2.3.2 MRI Imaging Session (Session 2)

**Figure 1:**
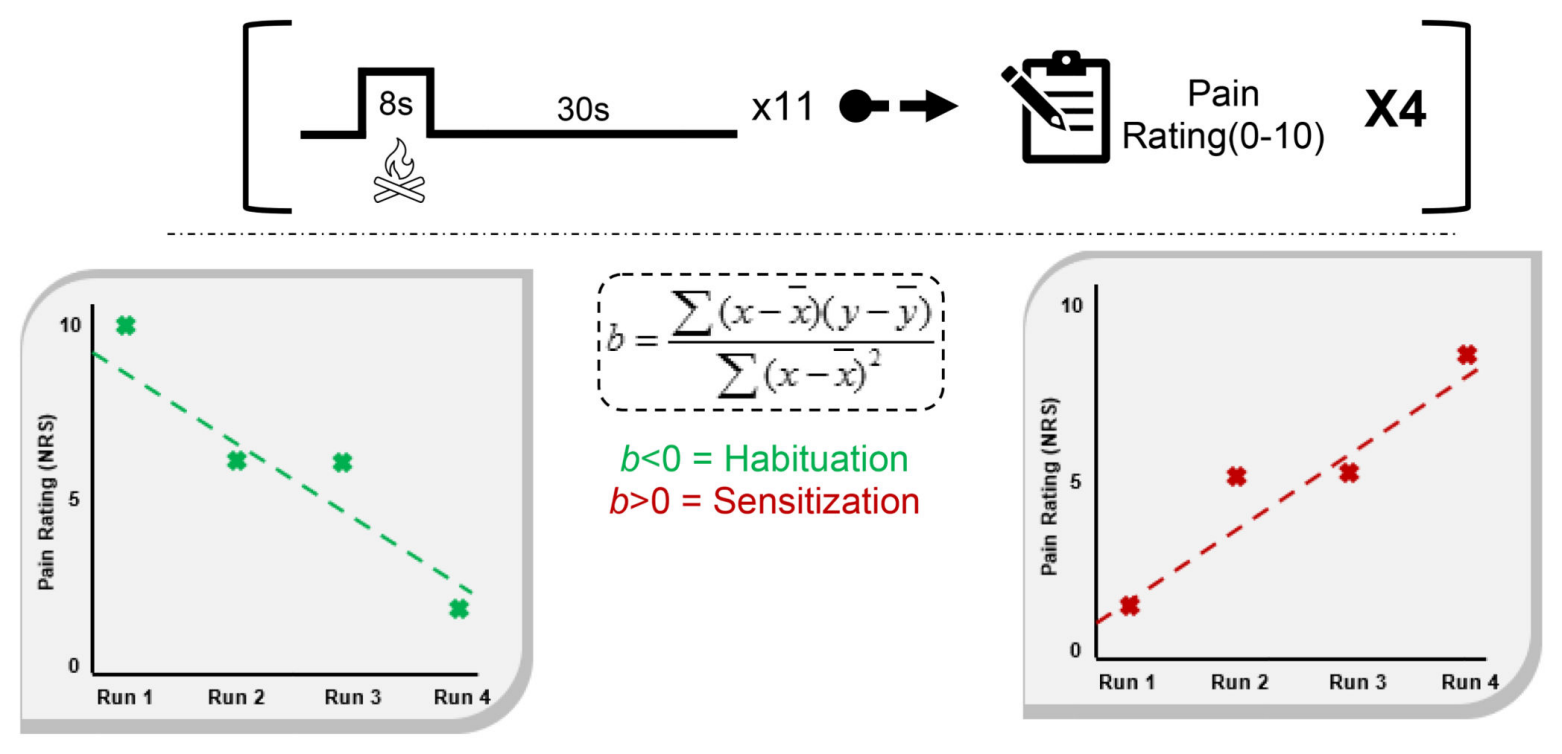
Experiment Design | Each block(top) consisted of 11 thermal stimuli. After the stimuli were administered, participants were asked to provide an average pain intensity rating for the stimuli that preceded. After four blocks, a linear regression equation was fitted to the points and an angle of slope was calculated. The quantification of pain habituation/sensitization was based on this slope, with a negative value representing habituation(bottom-left), and a positive value for sensitization(bottom-right). Data presented in these graphs is hypothetical for explanatory purposes, with block number on the x-axis, and with the provided pain rating for each block on the y-axis.

Participants attended an MRI session shortly after completing their baseline assessment (Mean=2.4 days, range= 1-11 days). Participants were instructed to keep their body and, specifically, their head still. They were also instructed to keep their eyes open and to focus on a fixation cross projected via a monitor and mirror attached to the head coil. After an initial localiser scan, structural images were acquired using a 5min 21s T1 anatomical scan, which was followed by an 8min 10sec resting-state scan. Participants then received the task instructions a second time and were given a test stimulus to their left leg, using the calibrated temperature for pain habituation/sensitization quantification, from the baseline assessment, to recheck tolerability (which all participants confirmed). The pain habituation/sensitization task (Fig.1) was divided across four EPI scans. Each block consisted of 11 stimuli based on the intensity calibrated in the previous session (6-8 NRS), with a duration of 8s, ramp and return rates of 8°C/s and an interstimulus interval (ISI) of 20s. At the end of each block, participants were asked to provide an average pain rating of the preceding stimuli, using a 4-button response pad. Participants were instructed to tap the furthest left-hand button to move the rating slide to the left, and vice versa for the far-right button. The two spin-echo field maps were then collected to finish the scanning session.

#### 2.3.3 Pain Stimulation Sessions (Sessions 3-5)

The following three sessions were completed following the imaging session (Mean=3.9 days, range= 1-9 days). These pain stimulation sessions consisted of psychometric assessment, wherein participants completed questionnaires at the beginning of each session. After this, participants completed the same task as they received in the MRI scanner (Fig.1), with the thermode applied to their left calf. As such, each participant received four blocks of a series of 11-stimuli, providing an average pain intensity rating at the end of each block. The stimulus temperature used for nociceptive stimulation was kept stable across the MRI and these three behavioural sessions.

### 2.4 Data Reduction and Analysis

#### 2.4.1 Behavioural Data Analysis

##### 2.4.1.1 Quantification of Habituation/Sensitization Slope and Temporal Summation

Previous studies have identified weaknesses in simply characterising habituators and sensitizers via dichotomous splits, as this approach forces participants with very little change into a distinct bipolar comparison[57,88]. Therefore, in this study, habituation/sensitization was quantified as a continuous variable. For each participant, we calculated the slope of a linear fit across the pain ratings provided at the end of each of the four blocks of pain stimulation. The design, timings and stimulus intensity were identical across the four sessions (Fig.1). For the calculation of the slope, the four pain ratings were plotted alongside an absolute linear pattern (1,2,3,4), facilitating the plotting of a regression line. As can be seen in Figure 1, this entails that if pain ratings increase over the runs, a positive slope value would be returned, representing sensitization. Whereas ratings decreasing over time would produce a negative slope value indicating habituation. Therefore, this process produced a separate habituation/sensitization slope for sessions 2, 3, 4 and 5 for every participant. In line with this approach, TS was also calculated as a slope value using the same approach as with habituation/sensitization slopes.

##### 2.4.4.2 Behavioural Data Analysis

To ascertain that the habituation/sensitization slopes across all sessions represented a stable measure of individual differences, the intraclass correlation(ICC) of the slopes across sessions 2, 3, 4 and 5 were calculated. Further, to evaluate the influence of contextual experimental effects (MRI vs lab-based assessments of pain habituation), ICCs were also calculated for sessions 3-5, which were all completed in the same lab. In the instance that ICCs indicated stability of slopes, an average slope was calculated, to evaluate correlations across baseline assessment variables and to evaluate the predictability of MRI pain rating behaviour. In line with recommendations for the implementation of multiple comparisons correction for neuropsychological data, the Hochberg’s modified Bonferroni method was applied[15]. All data were analysed using SPSS27(IBM Corp., Armonk, NY). Lastly, to investigate temporal sensitivity in pain across the course of the whole experiment, intrasession pain ratings were averaged and repeated measures ANOVAs were completed to statistically evaluate the extent of change over time. In the case of statistically significant effects, post-hoc paired comparisons were completed to ascertain the localisation of effect in time.

#### 2.4.2 MRI Data Analysis

##### 2.4.2.1 Pre-processing

FSL6.0[39] was used for all MRI analyses, including pre-processing, adhering to the protocol described by the CompCor[10]. Skull-stripping was performed using the Brain Extraction Tool (BET;[86]). Data were spatially smoothed using a 5mm FWHM Gaussian kernel, and interleaved slice timing correction was applied. To correct for B0 inhomogeneities in the data, the SE EPI acquisitions with opposite encoding directions were used to calculate a field map via TopUp[4] and applied to data. Functional data were registered to each subject’s anatomical space via Boundary-based registration (BBR) and registered to standard MNI space using 12-DOF non-linear transformation.

Using FAST[102], anatomical images were then segmented into grey matter, white matter (WM) and cerebrospinal fluid (CSF) masks. Masks were thresholded at .99, residuals were bandpass filtered (0.1/0.01Hz), normalised and time series for WM and CSF were generated. Using MCFLIRT[38], motion correction was applied, and this time-series was entered into a ‘nuisance removal’ GLM alongside WM and CSF time-series.

##### 2.4.2.2 Functional MRI Pain Task

The pre-processed single-subject data were modelled using pain stimulation events as explanatory variables, and then each of the four blocks of the pain task were concatenated using a fixed effects model as the second step. To capture changes in neural processes over time, alongside univariate concatenation (1,1,1,1), the data were also parametrically modulated in this second level analysis, to generate contrast maps for linear increases (-1.5, -0.5,0.5,1.5) or decreases (1.5,0.5, -0.5, -1.5) in activity across the blocks. All FEAT directories were entered into a third-level mixed effect FLAME1+2 analysis, wherein group mean, and habituation/sensitization slope (within the MRI task) were entered as explanatory variables. In addition to these variables, to ensure that the variation in stimulus temperature is appropriately controlled for, mean-centered stimulus temperatures were also added to the model. Parameter estimates of significant clusters were extracted using FeatQuery. For post-hoc analyses, when large clusters encompassed multiple regions, probabilistic anatomical masks were thresholded at 50% probability, and used to extract values within the cluster specific to an anatomical region. Temporal dynamics in activity over the four runs were quantified by calculating the slope angle of parameter estimates over time. These neural slopes were then entered into separate linear regression models to predict single-session temporal summation scores as well as pain rating slopes calculated across the pain ratings obtained in sessions 3, 4 and 5. Lastly, these neural slopes were correlated with the auxiliary psychosocial variables, to better conceptualise the processes that may be associated activation changes in these brain regions. Raw MRI co-ordinates within MNI space and statistical values at cluster peaks are provided in supplementary D, and the graphical representation of parametric modulation analyses can be found in supplementary E and G

##### 2.4.2.3 Resting-state MRI

In order to examine the connectivity patterns that might support regions associated with individual differences in habituation/sensitization to pain, clusters originating from the analysis of the functional pain task were used as ROIs in a resting-state psychophysiological interaction (PPI) analysis. Using the same cluster masking procedure employed for parameter extraction in the previous analysis, larger clusters which encompass multiple regions were masked with anatomical ROIs, to assess functional specificity of distinct anatomical regions within these large clusters. Psychophysiological interactions were investigated by extracting mean time series for regions-of-interest (significant clusters from the fMRI pain task), which were included as predictors in a whole-brain connectivity analysis. Contrast maps were then entered into a higher-level analysis with demeaned habituation/sensitization slopes, derived from MRI task pain ratings, as between-subjects regressors. This resulted in regions whose connectivity with the seed region was significantly correlated with pain habituation/sensitization. Analyses were corrected for multiple comparisons using Gaussian random field theory (z>2.3, p<.05). Using FeatQuery, parameter estimates were extracted from clusters showing individual differences in functional connectivity with each ROI as a function of habituation/sensitization slopes. To restrict the extracted data to grey matter regions, each cluster was multiplied using masks within the Harvard-Oxford anatomical atlases, using a 25% probabilistic threshold. To ascertain the psychological relevance of these brain areas, correlations were completed between entire clusters, and their anatomical subdivisions, and the psychosocial variables described in the behavioural analysis. Raw MRI co-ordinates within MNI space and statistical values at cluster peaks are provided in supplementary D.

### 3.1 Results

#### 3.1.1 Pain Habituation/Sensitization

**Figure 2:**
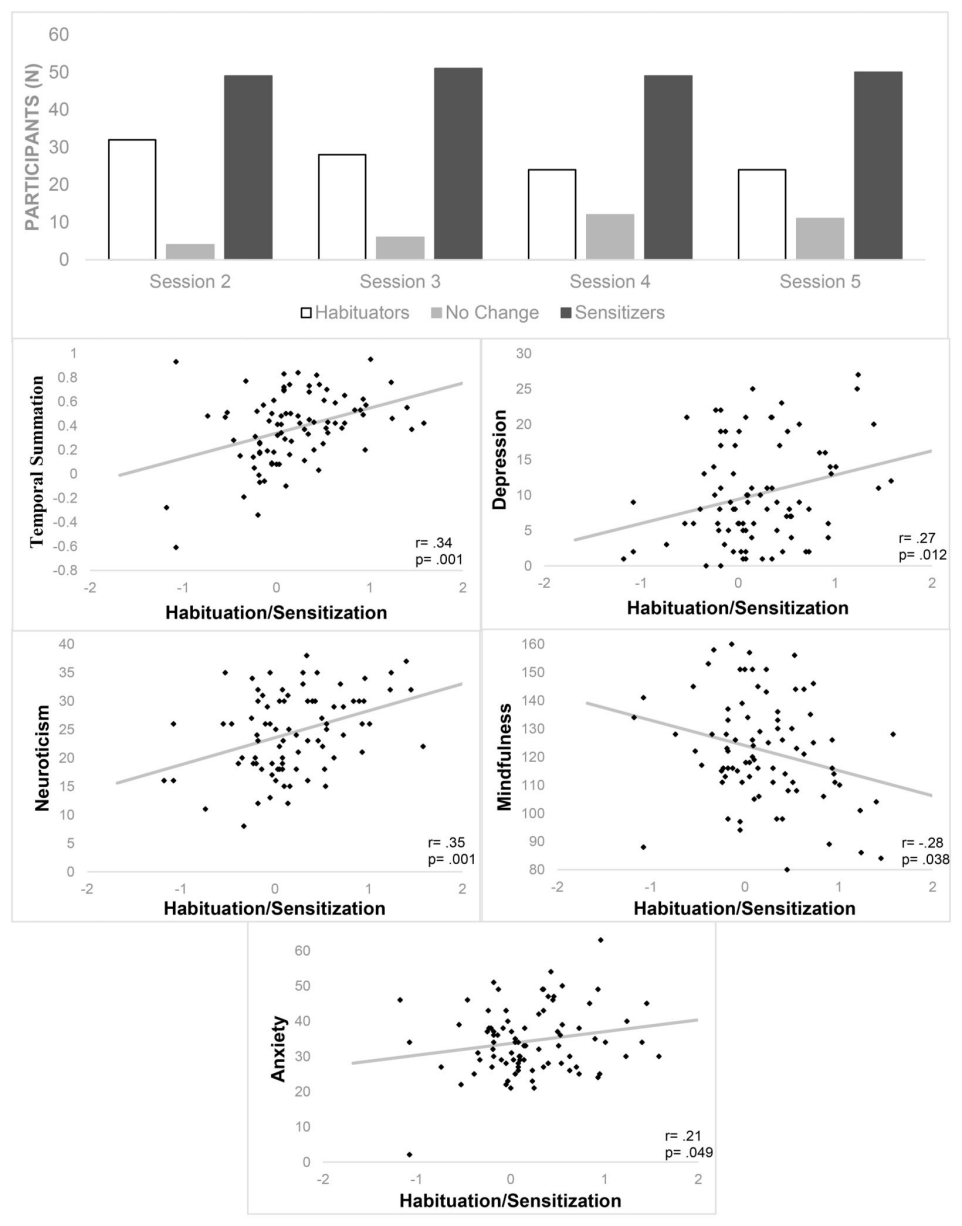
**a** (top) | Number of participants within each session that habituated, sensitized, or showed no change to repetitive painful stimulation. Figure 2b (bottom) | Correlations between average pain habituation/sensitization slope across all four sessions (higher value; predisposition to sensitization), and baseline psychobehavioural variables.

Across the sample, 37.6% (n=32) of participants demonstrated overall habituation to painful stimulation during the MRI task, as opposed to 57.6%(n=49) who sensitized, and 4.7% (n=4) who showed no change(Fig.1a;raw values in supplementary A). The average temperature of stimulus temperature within the pain adaptation task was 41.2°C (s.d=1.29,41.2°C-49°C), with the average temporal summation temperature 45.1°C (s.d=1.88,38.4°C-48.9°C). Pain rating slopes were stable across all four sessions (ICC(2,1)=.47(.36-.58), p<.001; Fig.2). The data indicated the presence of context effects on reported pain over time, represented by higher stability when excluding the MRI session (ICC(2,1)=.64(.57-.74),p<.001). As the slopes across sessions 3-5 were shown to be stable, a single average behavioural slope value was calculated and correlated with relevant variables obtained during the baseline sessions. Pain habituation/sensitization during the MRI task was significantly correlated with average slope in the following sessions (r(85)=.32,p=.003). Following multiple comparisons correction, those with higher predisposition to sensitization showed higher temporal summation (r(85)=.34,p=.001), neuroticism (r(84)=.35,p=.001), depression (r(85)=.27,p=.012) and anxiety scores (r(85)=.21,p=.049) (Fig.2b).. The Hochberg’s descending correction approach utilises a diminishing critical p-value correction from smallest-to-largest p-value. Therefore, despite not being the largest p-value, the correlation between mindfulness did not survive multiple comparisons correction (r(85)=-.23,p=.038). Habituation/sensitization slope was also correlated with the stimulation temperature itself (r(85)=.23,p=.034), potential indicating the influence of perceived risk of danger to the participant in their adaptation behaviour. Average intrasession pain ratings decreased over the four sessions (Supplementary B), and demonstrated a statistically significant effect (F(2.46,209)=25.16, p<.0001). Post-hoc paired comparisons identified the only significant reduction between sequential sessions was between sessions 2 and 3 (4.9 vs 4.0; p<.0001). Otherwise, significant effects were identified between sessions 1 and 3 (5.3 vs 4.0; p<.0001, sessions 1 and 4 (5.3 vs 3.8, p<.0001) and sessions 2 and 4 (4.9 vs 3.8, p<.0001).

#### 3.1.2 fMRI Pain Task

##### 3.1.2.1 Main Effects of Pain Stimulation

Pain stimulation activated brain areas frequently associated with pain, including bilateral insula, secondary somatosensory, premotor, and cingulate cortices, as well as the thalamus, bilateral amygdala, and brainstem. Parametric modulation analysis identified regions that demonstrated significant linear increases or decreases in activation during pain stimulation over the four blocks. Significant increases in activation were identified in the primary somatosensory and motor cortices, as well as in the parietal lobe and V1. Although, significant decreases in activation were identified in the right secondary somatosensory cortices, extending into the parietal operculum and Broca’s area, these did not survive correction following non-parametric permutation testing.

##### 3.1.2.2 Dynamic Mechanisms of Pain Habituation and Sensitization

**Figure 3:**
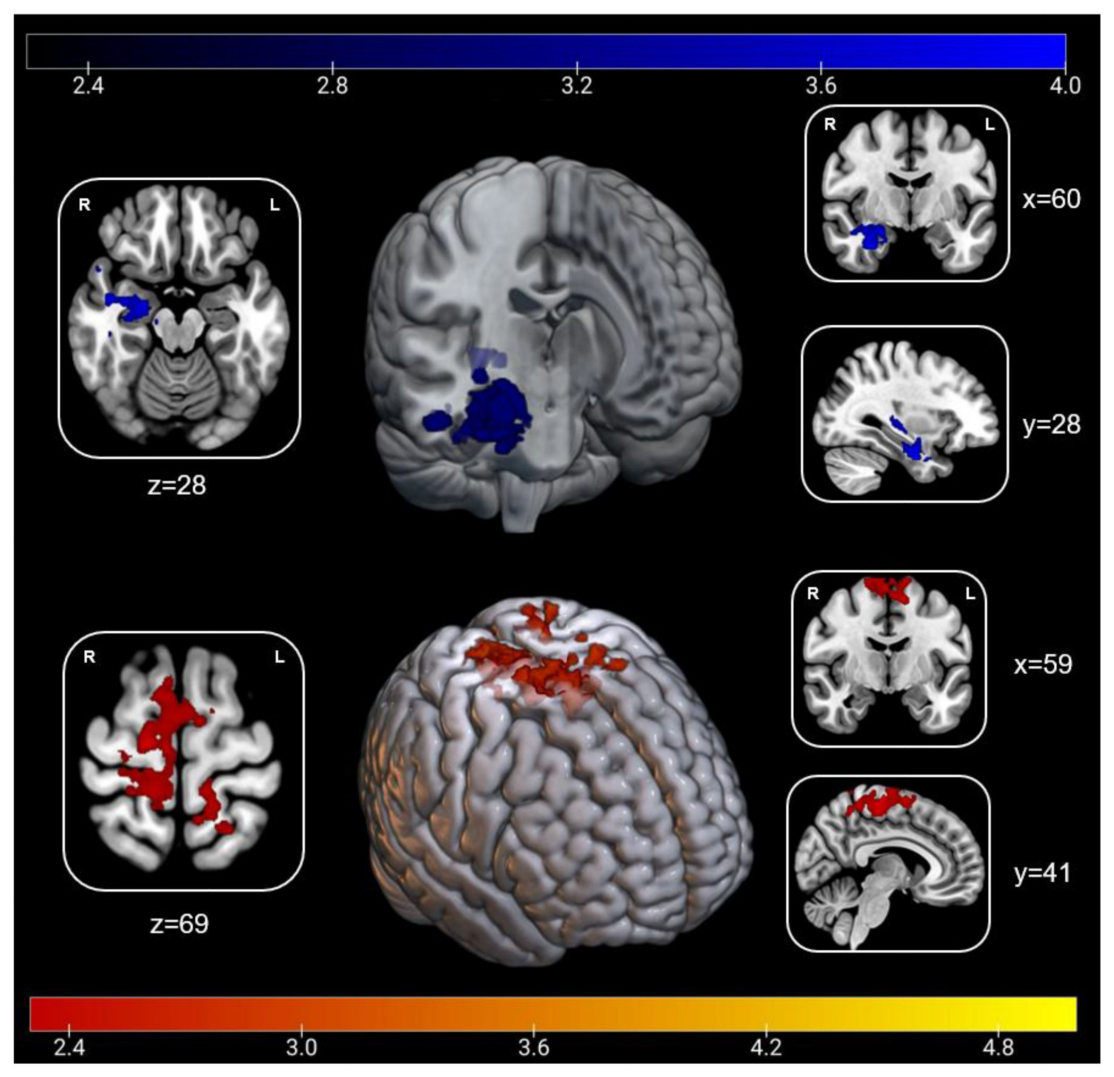
Dynamic mechanisms of pain habituation/sensitization across the scan session |(top) Cluster encompassing the right anterior hippocampus and amygdala (Peak Z_max_=3.97; x=28,y=-6,z=-30) where increasing activity is associated with degree of habituation to pain (bottom) Cluster encompassing the right premotor cortex and bilateral somatosensory and motor cortices (Peak Z_max_=4.49; x=6,y=-20,z=66) where increasing activity is associated with degree of sensitization. Numbers denote slice depicted.

Pain habituation/sensitization was associated with activity in the right anterior hippocampus and amygdala and right premotor cortex, bilateral primary somatosensory, and motor cortices across the four blocks (Fig.3). Such that, habituation was associated with increasing activity in the hippocampus and amygdala, and decreased activity in sensorimotor regions (Supplementary G). Parameter estimates were extracted from each cluster, and masked using probabilistic atlases (amygdala, hippocampus, somatosensory cortices and motor cortices). Activity change in the hippocampus over the four runs was significantly correlated with pain habituation/sensitization in the future behavioural sessions (sessions 3, 4 and 5) (r(85)=-.22,p<.05) and temporal summation scores (r(85)=-.25,p<.05) (Supplementary E). An increase in activity within the hippocampus was also associated with lower depression (r(85)=-23,p=.035) and neuroticism (r(85)=-.23,p=.033). Increasing activity change in the amygdala was correlated with lower neuroticism scores only (r(85)=-.26,p=.017). No significant correlations with psychosocial variables were identified for activity change in the sensorimotor cluster (Supplementary F).

#### 3.1.3 Resting-state MRI

##### Seed-based Whole-brain Functional Connectivity

**Figure 4:**
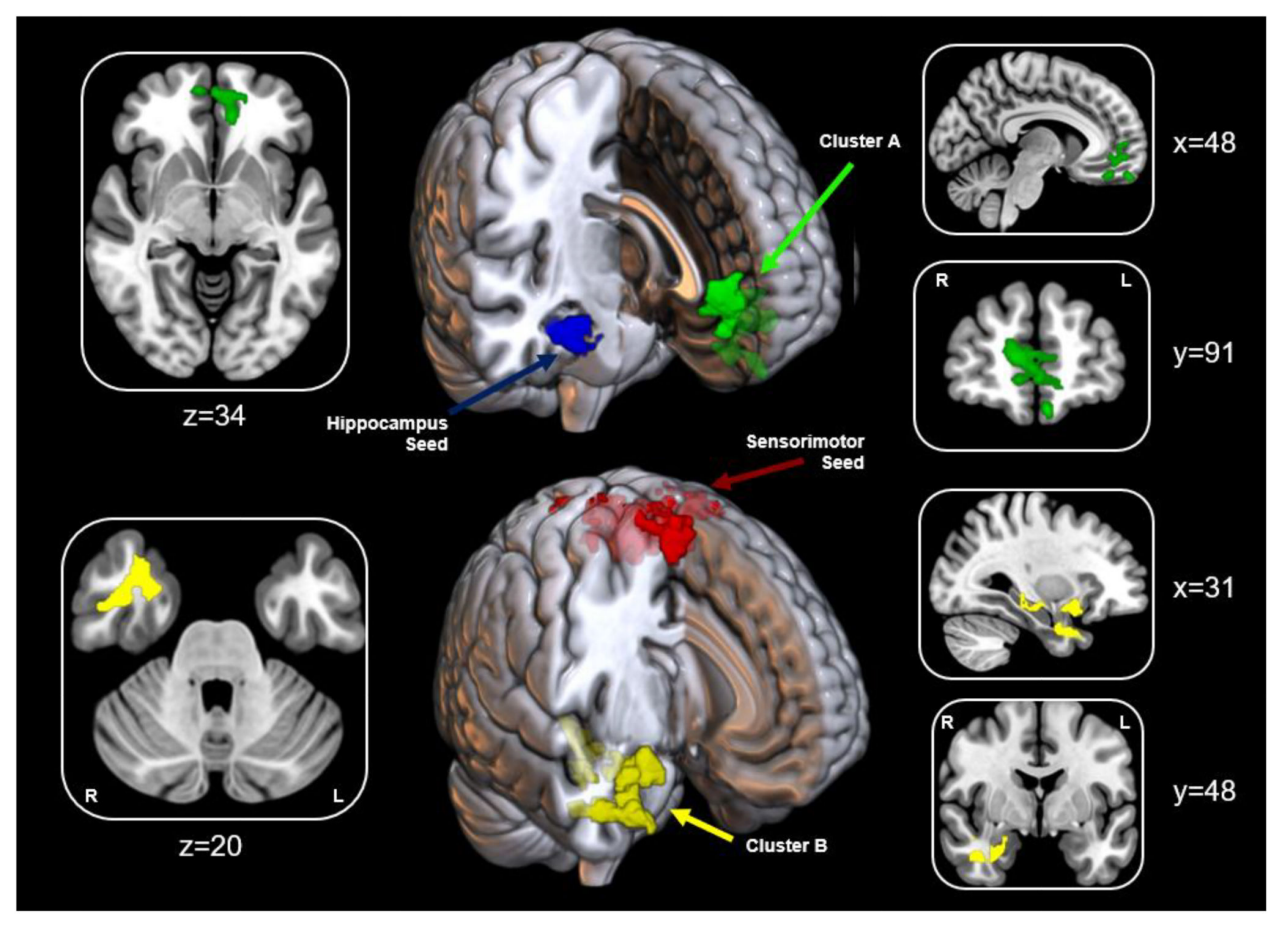
Functional Connectivity of Habituation |(top) Seed-based functional connectivity of the right hippocampus seed(blue) where habituation was associated with higher connectivity to the ventromedial prefrontal cortex(cluster A in green; Z_max_=3.78; x=-6,y=58,z=-22) |(bottom) Seed-based functional connectivity of the sensorimotor seed(red) where habituation was associated with higher connectivity to the right hippocampus, amygdala, and insula cortex(cluster B in yellow; Z_max_=4.3; x=30,y=12,z= -12). Numbers denote slice depicted.

To investigate the functional specificity of the two adjacent anatomical regions in the cluster resulting from the event-related fMRI analysis, connectivity analyses were performed using the cluster as a whole and the amygdala and hippocampus sub-clusters separately as seed regions. No significant results were identified when applying the amygdala as a seed, however, functional connectivity analyses revealed pain habituation/sensitization was associated with variations in connectivity between the right anterior hippocampus and ventromedial prefrontal cortex at rest, such that individuals with higher rates of habituation had higher connectivity between these regions (Fig.4). Habituation was also associated with higher functional connectivity between the sensorimotor cluster from the event-related analysis and a cluster extending across the right hippocampus, parahippocampal gyrus, amygdala, and insula cortex. This cluster partially overlapped with the cluster derived from the event-related habituation analysis. Resting-state analysis therefore yielded two connectivity pairings: Firstly, the hippocampus seed to ventromedial prefrontal cortex and, second, the sensorimotor seed to hippocampus. No significant correlations were found between the connectivity between either seed/cluster pairing and psychosocial variables (Supplementary F).

## Discussion

This study investigated the stability of individual differences in pain habituation/sensitization, and their underlying neural mechanisms. We observed substantial individual differences in response to repetitive nociceptive stimulation over time. Importantly, these individual patterns of pain habituation/sensitization were reliable across sessions, and experimental contexts. Habituation/sensitization slopes were also correlated with QST and psychometric variables, such that the degree of sensitization was associated with greater temporal summation, higher depression and neuroticism, and lower trait mindfulness. Higher levels of habituation were associated with increasing activity in the anterior hippocampus and amygdala over time, whereas sensitization was associated with increasing activity in the somatosensory, motor and premotor cortices. Activity change within the hippocampus over time was significantly correlated with, both, pain habituation/sensitization slopes in future behavioural sessions, and with single-session temporal summation. At rest, habituation was associated with higher functional connectivity between the hippocampus/amygdala and vmPFC, and higher connectivity between the sensorimotor cluster and the hippocampus, amygdala, and insula cortex. Collectively, our findings demonstrate that habituation or sensitization to pain involves a network of brain regions typically associated with the interpretation and evaluation of pain and is not solely a product of variation in nociceptive processing. As such, the way in which an individual processes pain is an important determinant of sensitization and habituation and, importantly, is a stable phenotypic quality.

How individuals respond to repetitive nociceptive stimulation is important to understanding vulnerability to pain. In circumstances where heightened risk is perceived, pain responses may be enhanced(sensitization). If unavoidable noxious stimuli are perceived to pose no threat, pain habituation could help preserve resources. When empirically examining group effects, proportions of habituators and sensitizers reported varies greatly[24,29,82,31,33,40,46,56,61,62,80]. Our finding is the first indicating the propensity for an individual to habituate or sensitize is stable within individuals across time and context and, hence, may serve as a valuable phenotypic marker.

### Sensory-discriminative habituation

Pain habituation was associated with reduced activity in the sensorimotor cortices. Reduced sensory-discriminative processing facilitating lower pain intensity is well documented[41,51] and this process appears to be disrupted in chronic pain patients[22,53]. Using a similar design, stimulation over 8 separate days was associated with reduced BOLD responses to nociceptive stimuli in SII, as well as in the insula, thalamus and putamen[12]. Those data also indicated that repeated painful stimulation was associated with increased grey matter density in the somatosensory cortex[91], which was maintained at 21-day follow-up. Importantly, most studies quantified habituation/sensitization exclusively by examining neural activity during nociceptive stimulation, without including pain ratings. Consequently, the neural results predominantly focus on sensory-discriminative regions and are likely solely tracking habituation processes related to reduced nociceptive processing, rather than higher order cognitive-affective modulatory processes. This current study advances our understanding of the subjective experience of pain habituation and sensitization by focusing on participant pain ratings, thus focusing on the phenomenological experience of pain, alongside the sensory dimension of nociception.

### Hippocampal-Amygdala Circuitry

Activity in the anterior hippocampus and amygdala was also associated with pain habituation/sensitization, with habituation linked to increasing activity over time. The interaction of amygdala-hippocampal processes are associated with the interpretation of emotional events and representations[76,84]. The anterior hippocampus is associated with pain information processing[48], mediation of context-dependent pain expectancy[104] and the integration of multiple sources of information to make decisions[101]. Chronic pain is associated with reduced bilateral hippocampal volume[103], disrupted hippocampal neurogenesis[25,52] and increased neuroinflammation[94]. Mirroring our resting-state findings, coupling between the hippocampus and mPFC has been identified as a biomarker for the transition from acute to chronic pain[55], with connectivity predicting variations in back pain across the year and likelihood of recovery from subacute pain, with persistent pain associated with large decreases in prefrontal-hippocampal functional connectivity[55]. Our finding advances this by highlighting that vmPFC-hippocampus connectivity in healthy controls predicts pain modulation, which may provide clarification to the mechanisms that predispose individuals to chronic pain. The underlying behavioural function of this biomarker could be pain habituation, with habituators best positioned for resolution of pain before it develops into a chronic condition. The expense of MRI prevents its utilisation in clinical pain assessment, making a behavioural proxy for a biomarker an asset. Whether this stimulation protocol could be applied to predict transition to pain chronicity requires further investigation.

The amygdala is also an important contributor to the emotional component of pain[32]. The latero-capsular division of the central nucleus of the amygdala, or “nociceptive amygdala”, is associated with the integration of external and internal environmental information with nociception[60,98]. Intuitively, the direction of this change in neural activity is surprising, given the relationship between the amygdala and pain intensity[16] and depressive or anxious states[3,23]. However, the amygdala is also involved in regulating moment-by-moment vigilance levels[96] and processing emotionally salient stimuli, especially when pertinent for later evaluation[20]. Within our design, participants were not informed their stimuli were all the same temperature provided their average pain intensity at the end of a trial. As pain involves an emotional component[77], our results suggest that the hippocampus and amygdala are involved in the consolidation of pain across repetitive stimulation via emotional appraisal processes. This mechanism may facilitate accurate appraisal that stimulation intensity was not increasing or risking physical damage, thus reducing threat appraisal and, ultimately, facilitating a beneficial habituatory response.

### Pain Habituation/Sensitization as a Phenotype

The influence of pain sensitization on pain chronicity is an crucial area of study[6,63], focusing on the discrepancy between peripheral drivers of pain, and the perceptual consequences. Pro-nociceptive phenotypes have long been investigated to identify those at risk of chronification of pain or poor treatment response[99]. Psychometrics, such as the central sensitization inventory(CSI), may be used for this purpose[59], although it is unclear if CSI actually quantifies its namesake construct[2]. Instead, QST is a more appropriate tool for quantifying these profiles[1,17,42], with temporal summation frequently cited as a potential tool for prediction [8,72,95]. In our data, TS was correlated with habituation/sensitization slopes and hippocampal activity dynamics. Taken together, this repetitive pain stimulation protocol may be a useful method for quantifying nociceptive phenotypes.

Habituation may represent an individual appropriately evaluating their environment as posing no risk of harm, facilitating the reduction of needlessly expended defensive resources. Sensitization to pain elicits the opposite and poses a risk of cognitive drain and increased pain sensitivity. Our data indicate that psychosocial features influence an individual’s predisposition towards either dimension. Neuroticism is a trait commonly associated with exaggerated threat-appraisal, and sensitivity to environmental stress[97]. Neuroticism is consistently associated with the transition from acute to chronic pain[71], the development of chronic post-surgical pain[30], impaired quality of life[78] and is evaluated in chronic pain sufferers as compared to healthy control[43]. Relatedly, depression is highly co-morbid with pain[81], and associated with poor clinical outcomes[64,74], pain-related interference and disability[47]. Within our data, both neuroticism and depression were correlated with habituation/sensitization slopes, with higher scores facilitating sensitization. Conversely, mindfulness, which was associated with pain habituation, is characterised as a non-judgemental present-moment attentional regulation[14], associated with lower pain sensitivity[36] and higher functioning in chronic pain patient[49]. While this study lacks of a measure of pure peripheral function that could clarify the involvement of peripheral mechanisms (such as nociceptor fatigue or sensitisation), the data suggest that pain habituation/sensitization is a process that extends beyond only nociceptive sensory pathways, involving higher-order cognitive and emotional responses as well. When considering that pain rating slope was correlated with the individualised stimulus temperature, this supports the position that heightened perception of threat of harm could predispose an individual to a greater risk of sensitisation. Functional connectivity analyses at rest further indicate that participants with decreasing sensorimotor activation during the task, who habituated to pain, showed increased connectivity with regions of the brain associated with emotional responding and learning(hippocampus, amygdala and insula). In turn, these regions were increasingly coupled with the vmPFC, a key region for emotional regulation via flexible value assignment and the context-dependent inhibition of the amygdala[5,83]. This, not only, expands on this habituation/sensitization phenotype, but underscores the importance of emotional regulation in pain resilience and, by extension, psychological interventions to correct a pro-nociceptive phenotype.

### Summary

This study is the first to evidence the stability of individual differences in pain habituation/sensitization and underscore its utility as a phenotypical variable, potentially suitable for clinical use. How a participant rates pain following repetitive nociceptive stimulation in a single session can predict similar rates of change in subsequent sessions. Individual differences in habituation/sensitization are stable across multiple sessions, and are associated with temporal summation, depression, neuroticism, anxiety and mindfulness. Reduced sensory-discriminative and increased amygdala-hippocampal activity is associated with pain habituation. Further, it is likely that functional connectivity between the hippocampus and the insula and vmPFC are similarly associated with habituation. Our data suggest that how an individual habituates or sensitizes to repetitive nociception involves, both, reductions in sensory nociceptive signals and higher order evaluative processes, which include the appraisal of pain and the regulation of emotion. Future work is needed to explore if these neural mechanisms represent biological markers for vulnerability to prolonged nociceptive input or whether a repetitive pain stimulation paradigm can be used as a phenotypical assessment to predict pain chronicity.

## Supplementary Material

Supplementary A

Supplementary B

Supplementary C

Supplementary D

Supplementary E

Supplementary F

Supplementary G
