# Supplementary material for: Stable individual differences in habituation and sensitization to prolonged painful stimulation are underpinned by activity in the hippocampus, amygdala and sensorimotor cortices
Source: Pain. Author manuscript; Available in PMC 2026 Jan 21. (PMC7618661; doi:10.1097/j.pain.0000000000003636)
Supplement: Supplementary A [file EMS211975-supplement-Supplementary_A.pdf]

Supplementary File A: Overview of demographics of participant sample

Characteristic	Value	Range
Age in years, mean (SD)	23.4 (5.48y)	18-45y
Gender, female, n (%)	44 (51.8%)	NA
Pain Threshold (°C), mean (SD)	44.6 (1.88°C)	37.9°C- 48.4°C
Temporal Summation, mean (SD)	4.8 (3.07)	-5 – 10
Depression score (BDI), mean (SD)	10.1 (6.97)	0 – 27
Neuroticism (BFI), mean (SD)	24.5 (6.92)	8 – 38
Trait Mindfulness (FFMQ), mean (SD)	122.33 (18.24)	80 – 160
Anxiety (STAI), mean (SD)	34.33 (9.38)	2.1 – 63
Session 1 (MRI) pain intensity (/10), mean (SD)	5.3 (1.80)	1-9
Session 2 pain intensity (/10), mean (SD)	4.9 (1.95)	0.25-8.75
Session 3 pain intensity (/10), mean (SD)	4.0 (1.85)	0-9
Session 4 pain intensity (/10), mean (SD)	3.8 (1.93)	0-8.75
