# Supplementary material for: Stable individual differences in habituation and sensitization to prolonged painful stimulation are underpinned by activity in the hippocampus, amygdala and sensorimotor cortices
Source: Pain. Author manuscript; Available in PMC 2026 Jan 21. (PMC7618661; doi:10.1097/j.pain.0000000000003636)

Supplementary File B: Individual pain habituation/sensitization slopes across all four repetitive painful stimulation sessions.

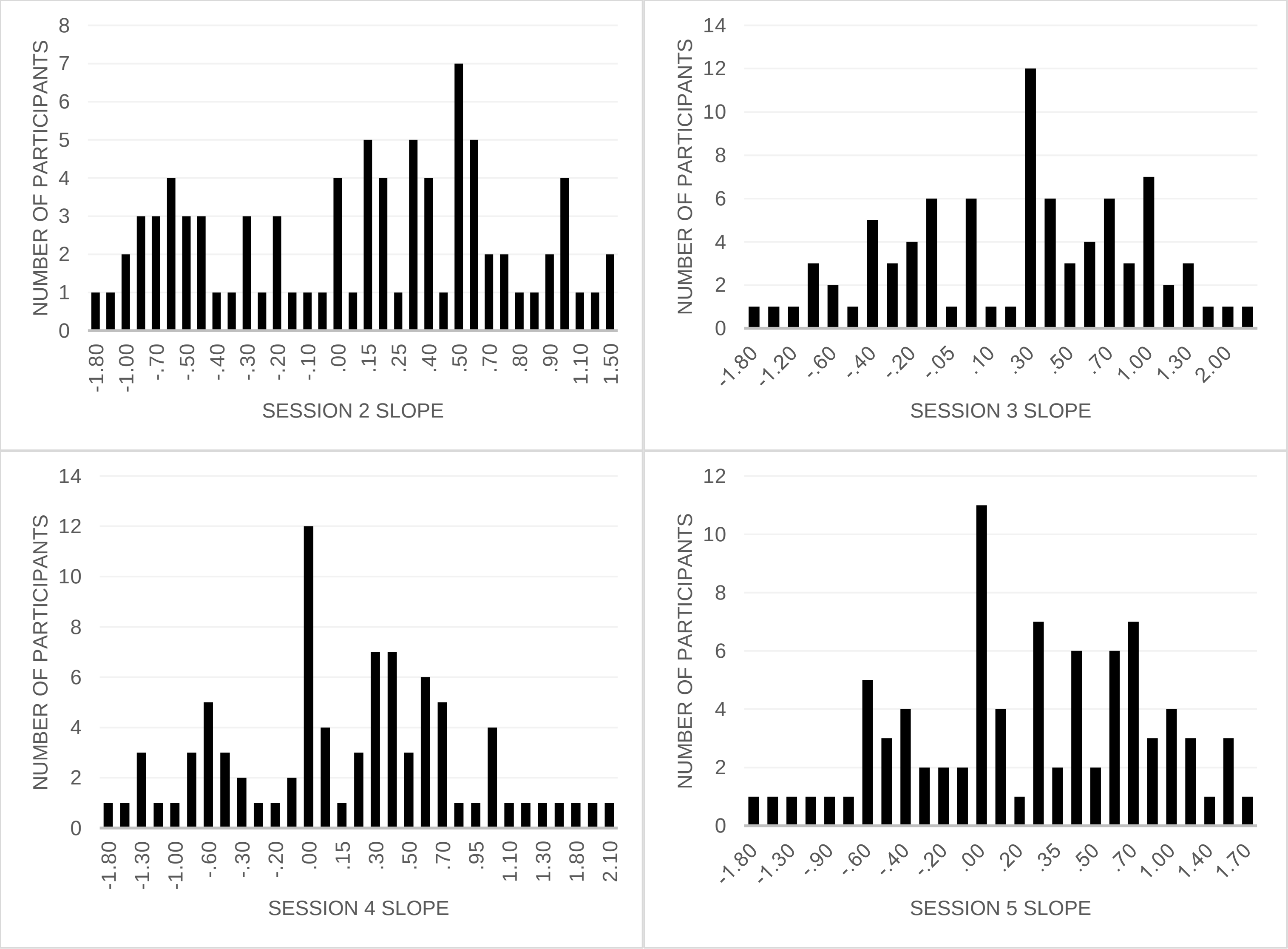

Supplement: Supplementary B [file EMS211975-supplement-Supplementary_B.pdf]
