# Supplementary material for: Stable individual differences in habituation and sensitization to prolonged painful stimulation are underpinned by activity in the hippocampus, amygdala and sensorimotor cortices
Source: Pain. Author manuscript; Available in PMC 2026 Jan 21. (PMC7618661; doi:10.1097/j.pain.0000000000003636)
Supplement: Supplementary C [file EMS211975-supplement-Supplementary_C.pdf]

Supplementary File C: Multiple regression for the prediction of average behavioural habituation/sensitization slope, with part and partial correlations

R	R^2	Adjusted R^2
.531	.282	.236

Variable	Standardised β	t	Sig.	Correlations		
				Zero-order	Partial	Part
Model constant		-1.534	.129			
TS	.386	3.970	0.00015	.369	.410	.381
STAI	.152	1.436	.155	.213	.160	.138
BFI_N	.255	1.909	.060	.350	.211	.183
FFMQ	.002	.020	.984	-.225	.002	.002
BDI	.067	.560	.577	.271	.063	.054

Abbreviations: TS= Temporal Summation. STAI= State/Trait Anxiety Inventory. BFI_N= Big Five Neuroticism Subscale. FFMQ= Five Factor Mindfulness Questionnaire. BDI= Becks Depression Inventory.

Dependent Variable= Average habituation/sensitization slopes across sessions 3, 4 and 5.
