# Supplementary material for: Stable individual differences in habituation and sensitization to prolonged painful stimulation are underpinned by activity in the hippocampus, amygdala and sensorimotor cortices
Source: Pain. Author manuscript; Available in PMC 2026 Jan 21. (PMC7618661; doi:10.1097/j.pain.0000000000003636)
Supplement: Supplementary D [file EMS211975-supplement-Supplementary_D.pdf]

Supplementary File D: MRI co-ordinates of clusters reported in event-related and resting-state analyses. Co-ordinates presented in MNI space, with Max Z-stat at peak and anatomical brain regions associated with these peaks, as described by Harvard-Oxford anatomical atlases (cortical & sub-cortical)

**Anatomical Brain region**	**MNI coordinates**			**Max Z-Stat**
	X	Y	Z	
Frontal orbital & insula cortices (r)	30	28	4	11
Opercular & insula cortices (r)	36	6	12	10.2
Insula cortex (r)	36	-18	16	9.57
Opercular & insula cortices (r)	40	16	2	9.34
Opercular & insula cortices (r)	30	22	8	9.15
Operculum cortex (r)	52	0	6	9.05

Mean effects of pain

**Anatomical Brain region**	**MNI coordinates**			**Max Z-Stat**
	X	Y	Z	
Hippocampus and parahippocampal gyrus (r)	28	-6	-30	3.88
Amygdala (r)	24	-8	-16	3.52
Hippocampus (r)	32	-10	-22	3.5
Planum Polare (r)	44	-4	-18	3.48
Putamen (r)	32	-22	-2	3.34
Hippocampus (r)	34	-6	-18	3.27

Increasing activity across blocks of pain stimulation

**Anatomical Brain region**	**MNI coordinates**			**Max Z-Stat**
	X	Y	Z	
Postcentral gyrus (r)	12	-40	68	3.88
Supplementary motor cortex (r)	8	4	64	3.68
Superior frontal gyrus (r)	-12	-8	60	3.63
Supplementary motor cortex & precentral gyrus (r)	2	-10	70	3.63
Supplementary motor cortex (r)	-10	-12	60	3.62
Precentral gyrus (r)	4	-24	62	3.52

Decreasing activity across blocks of pain stimulation

**Anatomical Brain region**	**MNI coordinates**			**Max Z-Stat**
	X	Y	Z	
Frontal pole & paracingulate gyrus	-6	58	-22	3.78
Frontal pole & paracingulate gyrus	10	58	6	3.71
Frontal pole	12	60	10	3.47
Frontal medial cortex & paracingulate gyrus	-12	42	-4	3.35
Frontal medial cortex, frontal pole & paracingulate gyrus	-2	56	-6	3.2
Frontal medial cortex, frontal pole & paracingulate gyrus	2	56	0	3.16

Resting-state connectivity between hippocampus and medial prefrontal cortex

**Anatomical Brain region**	**MNI coordinates**			**Max Z-Stat**
	X	Y	Z	
Insula cortex (r)	30	12	-12	4.3
Insula cortex (r)	38	10	-16	4.21
Anterior hippocampal gyrus & Amygdala (r)	26	0	-28	3.84
Hippocampus and anterior hippocampal gyrus (r)	22	-32	-12	3.8
Insula cortex & amygdala (r)	30	6	-14	3.69
Temporal Pole (r)	32	6	-34	3.66

Resting-state connectivity between sensorimotor cortices and subcortical regions
