# Supplementary material for: Stable individual differences in habituation and sensitization to prolonged painful stimulation are underpinned by activity in the hippocampus, amygdala and sensorimotor cortices
Source: Pain. Author manuscript; Available in PMC 2026 Jan 21. (PMC7618661; doi:10.1097/j.pain.0000000000003636)

Supplementary File E |(top) Correlation between the change in hippocampal activity across the four runs of the pain stimulation task and behavioural pain rating slope in future behavioural sessions (sessions 3, 4 and 5). (bottom). The correlation between hippocampal activity across the four runs of the pain stimulation task and temporal summation quantified during the baseline assessment.

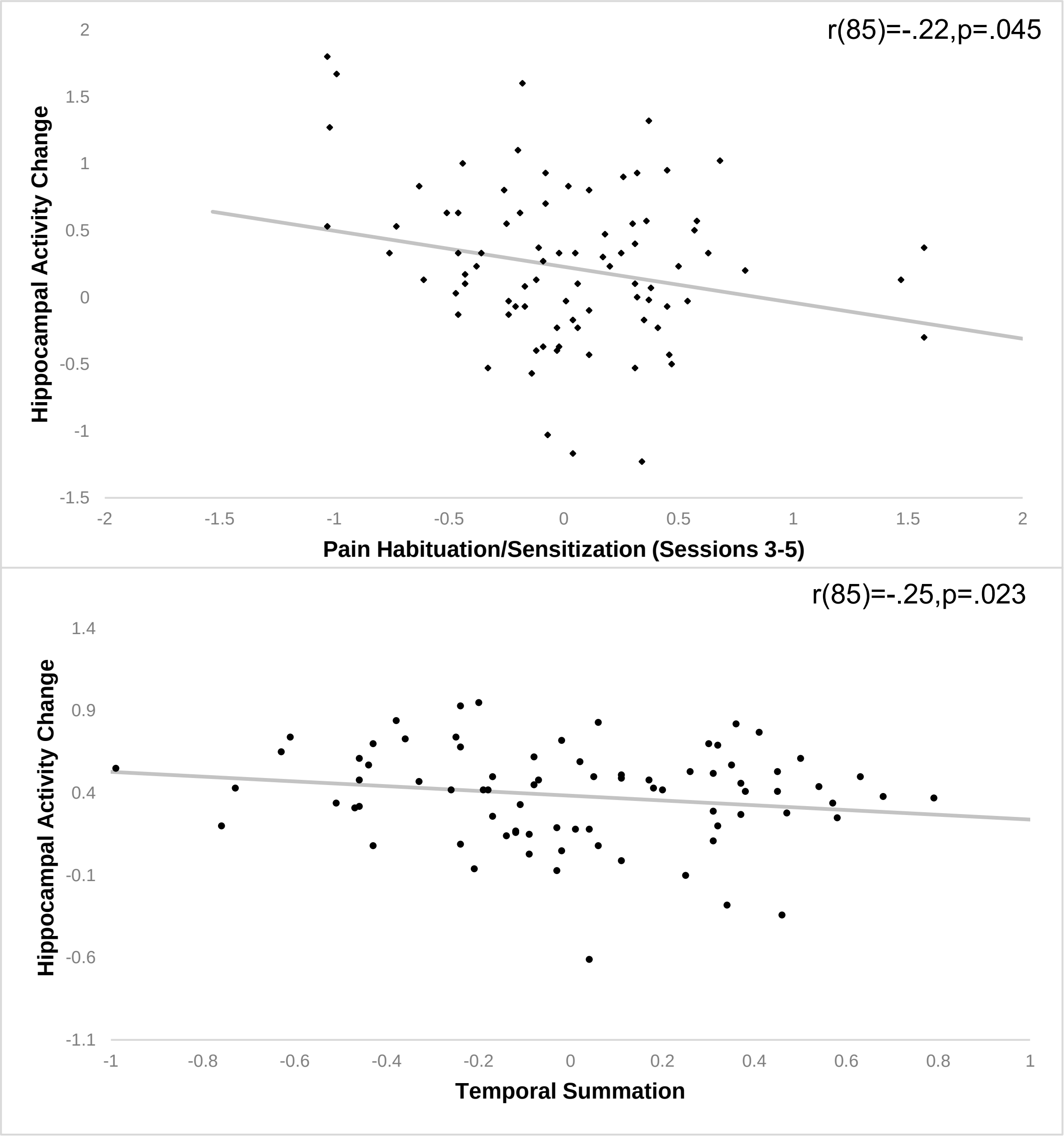

Supplement: Supplementary E [file EMS211975-supplement-Supplementary_E.pdf]
