# Supplementary material for: Stable individual differences in habituation and sensitization to prolonged painful stimulation are underpinned by activity in the hippocampus, amygdala and sensorimotor cortices
Source: Pain. Author manuscript; Available in PMC 2026 Jan 21. (PMC7618661; doi:10.1097/j.pain.0000000000003636)
Supplement: Supplementary F [file EMS211975-supplement-Supplementary_F.pdf]

Supplementary File F: Correlations between baseline psychosocial variables and results from event-related and resting-state MRI data

Variable	1	2	3	4	5	6	7	8
1. Hippocampal activity change	---							
2. Amygdala activity change	.82**	---						
3. Sensorimotor activity change	.01	.12	---					
4. TS	-.25	-.16	.17	---				
5. STAI	-.10	-.15	.03	-.10	---			
6. BDI	-.23*	-.16	-.11	.09	.30**	---		
7. Neuroticism	-.23	-.26	.08	-.01	.33**	.51**	---	
8. FFMQ	.10	.14	.07	-.04	-.27**	-.48**	-.53**	---

Supp F1: Correlations between activity change in regions identified via event-related MRI data, and psychosocial variables at baseline. All correlations corrected for multiple comparisons following Hochberg's modified Bonferroni method.

Abbreviations: TS= Temporal Summation. STAI= State/Trait Anxiety Inventory. BFI_N= Big Five Neuroticism Subscale. FFMQ= Five Factor Mindfulness Questionnaire. BDI= Becks Depression Inventory.

**. Correlation is significant at the 0.01 level (2-tailed)

*. Correlation is significant at the 0.05 level (2-tailed)

Variable	1	2	3	4	5	6	7
1. Hippocampus to mPFC	---						
2. Sensorimotor to hippocampus, insula & amygdala	.10	---					
3. TS	-.04	.04	---				
4. STAI	.03	-.04	-.10	---			
5. BDI	-.11	-.04	.09	.30**	---		
6. Neuroticism	-.20	-.08	-.01	.33**	.51**	---	
7. FFMQ	.15	.05	-.04	-.27**	-.48**	-.53**	---

Supp F1: Correlations between resting-state functional connectivity between seeds and identified whole-brain clusters, and psychosocial variables at baseline. All correlations corrected for multiple comparisons following Hochberg's modified Bonferroni method.

Abbreviations: mPFC= Medial prefrontal cortex. TS= Temporal Summation. STAI= State/Trait Anxiety Inventory. BFI_N= Big Five Neuroticism Subscale. FFMQ= Five Factor Mindfulness Questionnaire. BDI= Becks Depression Inventory.

**. Correlation is significant at the 0.01 level (2-tailed)

*. Correlation is significant at the 0.05 level (2-tailed)
