# Supplementary material for: Stable individual differences in habituation and sensitization to prolonged painful stimulation are underpinned by activity in the hippocampus, amygdala and sensorimotor cortices
Source: Pain. Author manuscript; Available in PMC 2026 Jan 21. (PMC7618661; doi:10.1097/j.pain.0000000000003636)

Supplementary File G | Correlations between the participant's pain habituation/sensitization slopes and the resulting contrast of parameter estimates (COPE) during the pain stimulation MRI task. Displayed are activity change within the sensorimotor cortices (top) and amygdala/hippocampus (bottom)

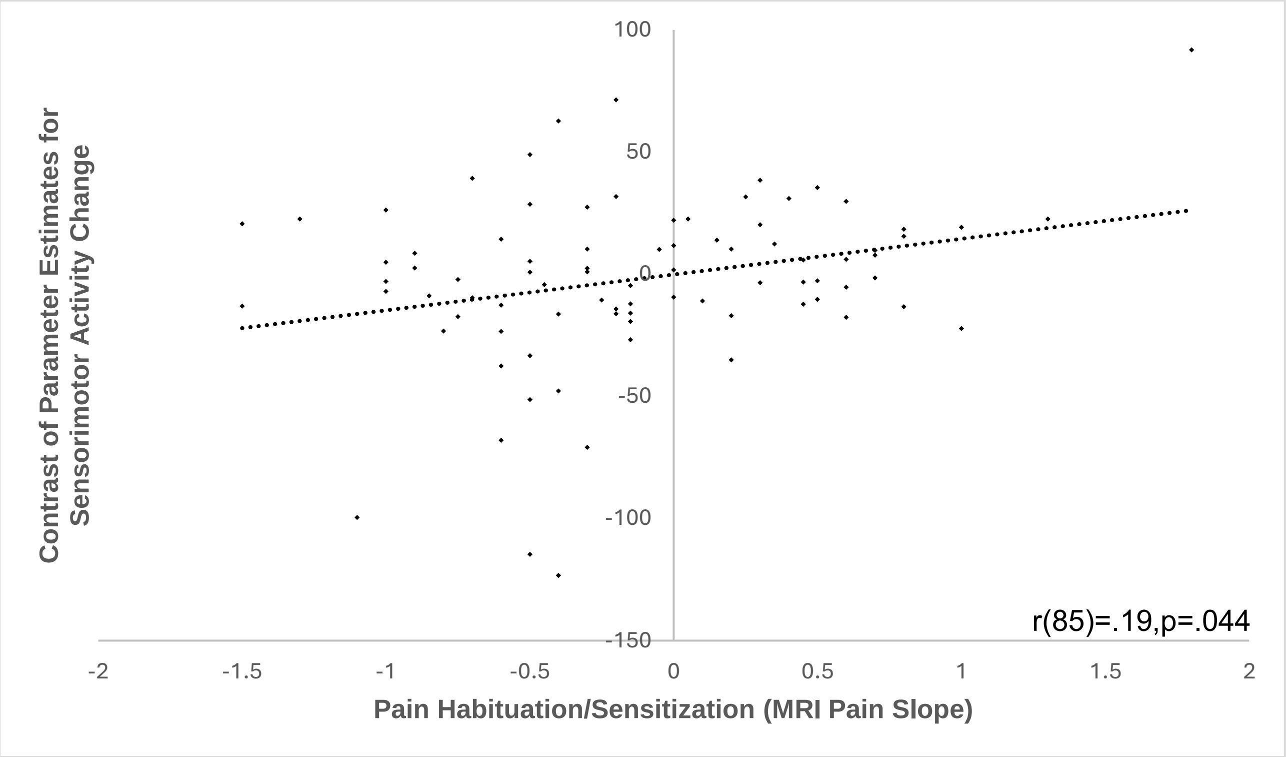

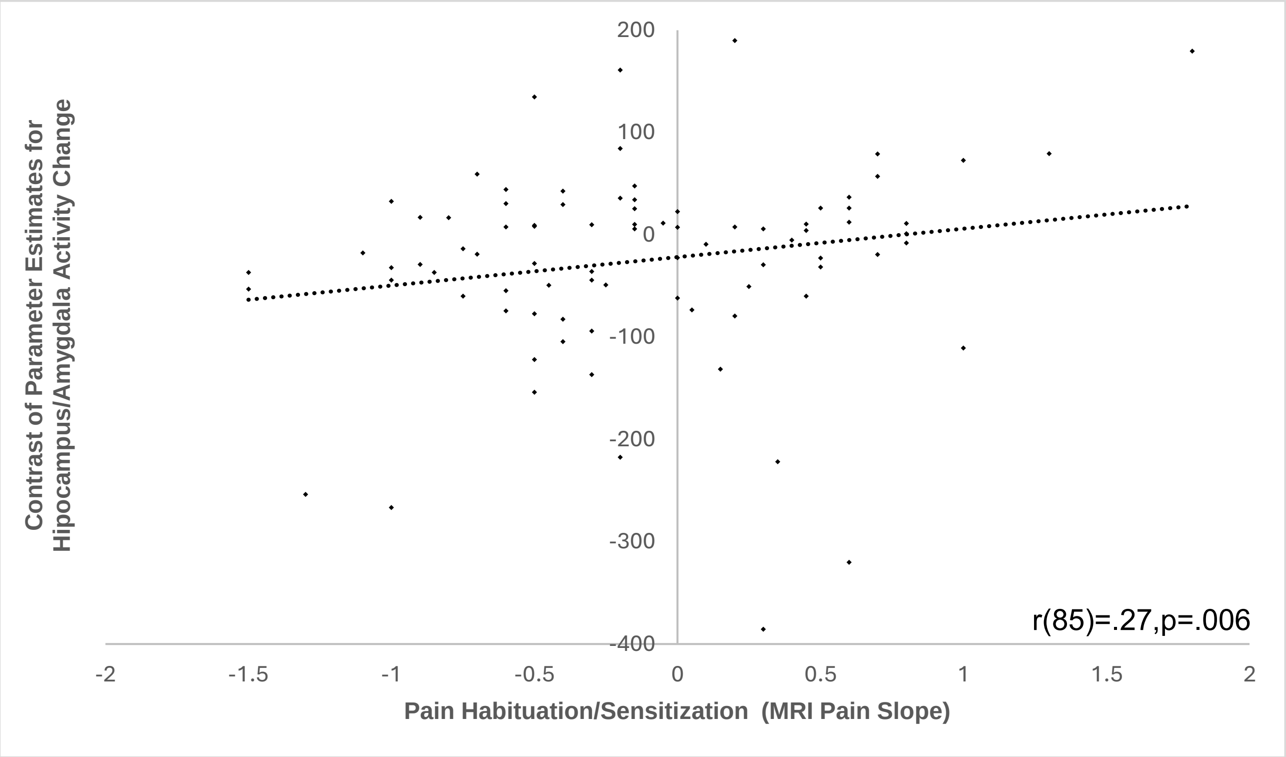

Supplement: Supplementary G [file EMS211975-supplement-Supplementary_G.pdf]
